# Body-Worn Sensors for Parkinson’s disease: A qualitative approach with patients and healthcare professionals

**DOI:** 10.1371/journal.pone.0265438

**Published:** 2022-05-05

**Authors:** Clara Virbel-Fleischman, Yann Rétory, Sébastien Hardy, Camille Huiban, Jean-Christophe Corvol, David Grabli

**Affiliations:** 1 Sorbonne Université, Brain Institute–ICM, Inserm, CNRS, Paris, France; 2 Air Liquide SA, Explor Center (Healthcare), Paris, Île‐de‐France, France; 3 Université Paris-Saclay, Laboratoire Complexité, Innovations, Activités Motrices et Sportives (CIAMS), Orsay, France; 4 Université d’Orléans, CIAMS, Orléans, France; 5 Institute of Memory and Alzheimer’s Disease (IM2A), Pitié-Salpêtrière Hospital, APHP, Paris, France; 6 Department of Neurology, Pitié-Salpêtrière Hospital, APHP, Paris, France; Yale University School of Medicine, UNITED STATES

## Abstract

Body-Worn Sensors (BWS) provide reliable objective and continuous assessment of Parkinson’s disease (PD) motor symptoms, but their implementation in clinical routine has not yet become widespread. Users’ perceptions of BWS have not been explored. This study intended to evaluate the usability, user experience (UX), patients’ perceptions of BWS, and health professionals’ (HP) opinions on BWS monitoring. A qualitative analysis was performed from semi-structured interviews conducted with 22 patients and 9 HP experts in PD. Patients completed two interviews before and after the BWS one-week experiment, and they answered two questionnaires assessing the usability and UX. Patients rated the three BWS usability with high scores (SUS median [range]: 87.5 [72.5–100]). The UX across all dimensions of their interaction with the BWS was positive. During interviews, all patients and HP expressed interest in BWS monitoring. Patients’ hopes and expectations increased the more they learned about BWS. They manifested enthusiasm to wear BWS, which they imagined could improve their PD symptoms. HP highlighted needs for logistical support in the implementation of BWS in their practice. Both patients and HP suggested possible uses of BWS monitoring in clinical practice, for treatment adjustments for example, or for research purposes. Patients and HP shared ideas about the use of BWS monitoring, although patients may be more likely to integrate BWS into their disease follow-up compared to HP in their practice. This study highlights gaps that need to be fulfilled to facilitate BWS adoption and promote their potential.

## Introduction

Parkinson’s disease (PD) is the second most common neurodegenerative disorder affecting about six million people worldwide [1]. In PD, the loss of dopaminergic neurons results in the three main motor symptoms that are the hallmarks of the disease: akinesia, resting tremor and hypertonia. Other symptoms, due to dopaminergic and non-dopaminergic lesions, include gait and balance deficits and a wide spectrum of non-motor symptoms. Currently, PD treatment is symptomatic and consists of dopaminergic substitution. Therapeutic strategies depend on the patients’ profiles and disease severity [2,3]. Although these strategies are personalized all along the care pathway, assessment of symptoms that could drive treatment adaptation, either during outpatients visits or hospitalizations, is highly challenging, particularly when motor fluctuations and dyskinesia occur [4]. A mismatch may exist between the physician’s assessment and the patient’s perception of his/her pathology [5]. This may be because of gaps in the patient’s knowledge of symptomatology or memory biases [6] or because of stress induced by the medical environment which may cause, for instance, a "white-coat effect" [7] that decreases the chances of observing specific symptoms. Among the different PD scales scoring the severity, the patient’s diary allows for an assessment of symptoms every 30 minutes [8]. Still, it is subjective, and compliance might be too low to provide reliable information [9]. In parallel, hospitalizations are costly and not ecological [10,11]. Facing these limitations, as suggested by Odin et al. [12], PD management could be improved by introducing Body-Worn Sensors (BWS) into PD care pathways as technology-based monitoring in ecological conditions.

BWS are wearable systems embedded with movement analysis algorithms to record the body’s activity. They are usually composed of triaxial accelerometers and sometimes triaxial gyroscopes, which capture displacement and speed and translate characteristics of movements into specific symptoms of PD. They allow continuous, quantitative and real-life monitoring of motor symptoms [13–15]. BWS can be worn on the wrist, the ankle, or the trunk, and can be used for several days in the patient’s home without medical supervision. Studies have shown that these systems provide relevant information on the driving states of PD in free-living conditions [16]. They can support clinical evaluation of the symptoms experienced daily to assess disease evolution and facilitate its understanding [17–21].

Ambulatory assessment of motor symptoms with BWS may be useful for PD management, although its clinical relevance is not yet demonstrated. However, more information is needed before fostering routine adoption of this technology [22]. Together with data regarding the clinical efficacy of BWS-based care, the assessment of the user experience (UX), from both the patients’ and healthcare professionals’ perspectives, is essential https://www.zotero.org/google-docs/?108Rgx[23]. Currently, only a few independent user-centered investigations have been conducted, and knowledge of the users’ opinions of BWS and their usability and attractiveness is still missing. This qualitative study intended to explore PD patients’ and health professionals’ (HP) perceptions of BWS technology, focusing on:

- BWS usability and patients’ UX
- PD patients’ opinion of BWS
- Neurologists’ and nurses’ opinion of BWS

## Methods

### Participants

Participants were recruited to take part in the study voluntarily. Participants signed an informed consent and they could withdraw from the study at any time, avoid answering a question, or stop the interview. The study focused only on participants’ perceptions and the patients included were followed in routine care. No data collected during this study was used to change the care delivered to the patients. The study took place from January to November 2019 and approval from the Review Board was waived.

#### Patients

Inclusion criteria for patients were a diagnosis of PD without dementia and their presence at the hospital once a week for routine care visits. Semi-structured interviews were conducted with patients who also completed two questionnaires at the end of their participation. Patients were divided into three groups according to the BWS worn.

**Fig 1:**
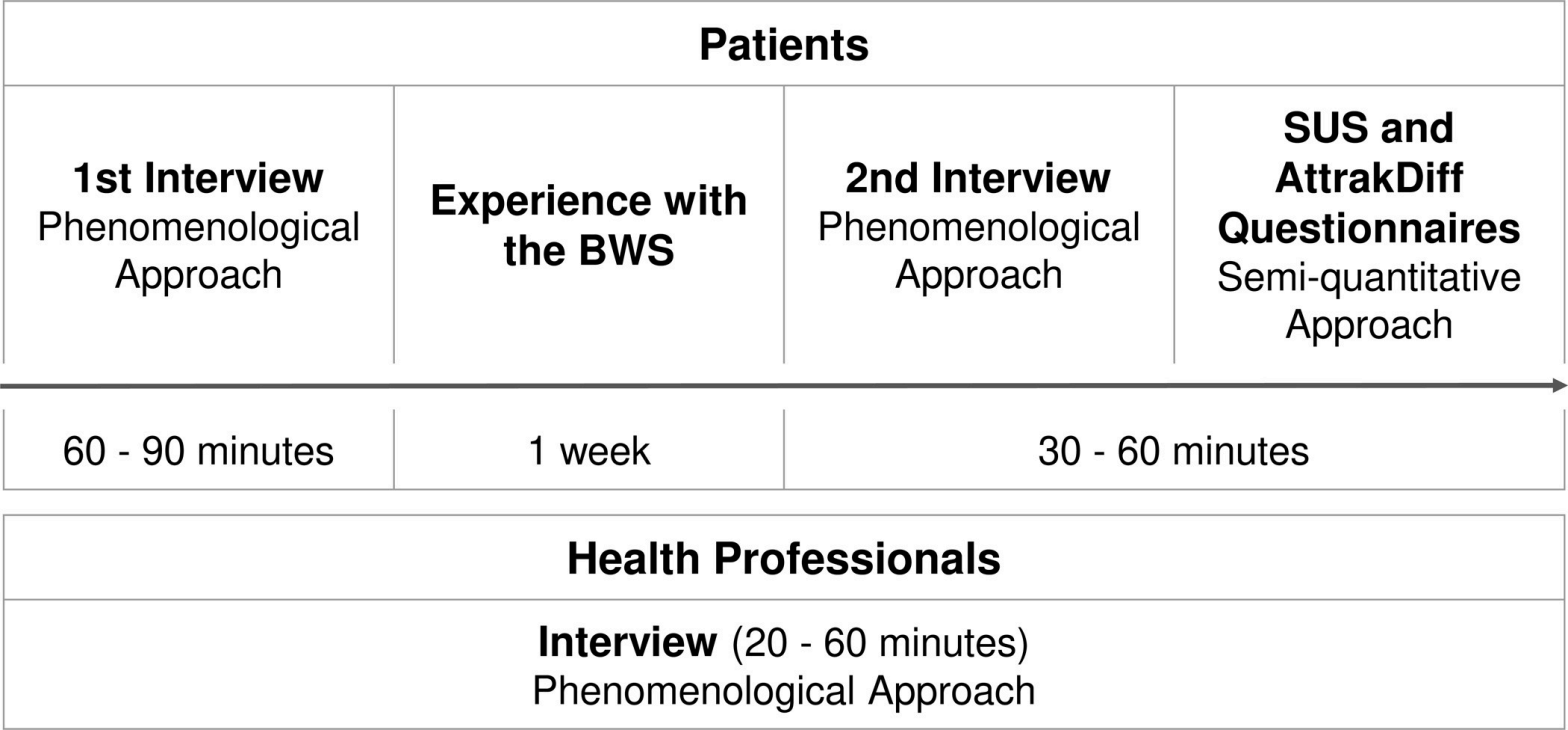
Participation protocol for patients and health professionals.

The procedure of participation included (Fig 1):

- the first contact at the hospital during a normal care visit for recruitment and interviews planning;
- the first interview, held at the hospital according to an interview guide, allowing patients to first handle one BWS, that was selected before interview depending on the BWS availability at the time of inclusion;
- a one-week experience with the BWS, at the patients’ homes without supervision; and
- the second interview, held at the hospital according to an interview guide and questionnaire completion.

#### Health professionals

Neurologists and nurses specializing in PD were selected from a listing of two Movement disorder expert centers in the Paris region to be invited to participate. The recruitment ceased when the sample size reached 9 HP. Semi-structured interviews were conducted with six neurologists and three nurses specializing in PD. The procedure for participation for those 9 HP included (Fig 1):

- the first contact by phone for recruitment and interview planning; and
- an interview held at the hospital according to an interview guide.

### Material

The selection criteria for BWS were the possibility to obtain objective and continuous monitoring of PD motor symptoms at the patients’ homes without medical supervision and the availability of sensors (CE mark) at the time of the study.

Three different BWS were selected for this experiment:

- BWS a: The PKG Watch (Global Kinetics Corporation, Australia) is a wrist-worn sensor installed on the most affected side of the body 24 hours per day. No specific handling is needed by the patients during home monitoring. Reports of symptoms show severities of bradykinesia, dyskinesia and motor fluctuations, and tremor and immobility presented as graphs and quantitative scores https://www.zotero.org/google-docs/?DZgBN9[24–27].
- BWS b: Kinesia 360 (Great Lakes NeuroTechnologies Inc., USA) is a set of two sensor bands worn on the wrist and the ankle of the most affected side of the body during waking hours. Once at home, the patient must install and remove the sensors and start and stop the monitoring every day, thanks to a dedicated Smartphone app. Sensors are charged, and data are downloaded each night. Reports of symptoms show probabilities of appearance and severity of slowness of movement, dyskinesia, tremor, as well as mobility data (*e*.*g*., time with gait, time at rest, arm swing percentage) numerically [28–31].
- BWS c: STAT-ON (Sense4Care, Spain) is a sensor-belt worn around the waist during waking hours. Patients need to install and remove it each morning and evening. The sensor is charged at night. The results of monitoring show a summary of motor symptoms with ON, OFF and intermediate states appearance across days as well as dyskinesia, freezing of gait and falls. An extended version of the report shows further details on motor states, mobility and gait parameters (*e*.*g*., stride fluidity, step length, cadence, energy expenditure) [18,32–34].

### Semi-quantitative approach: Usability and user experience of BWS to patients

The usability of BWS was studied with the System Usability Scale (SUS) [35]. The respondents rated their agreement with ten sentences on a 5-point Likert scale. The scores ranged from 0 to 100, with higher scores indicating greater agreement. The adjective ratings of Bangor et al. [36] was used to describe the categories of scores.

BWS UX was studied using the AttrakDiff questionnaire https://www.zotero.org/google-docs/?dGDhWQ[37,38], which assesses the interaction between the user and the product according to hedonic (HQ) and pragmatic qualities (PQ) described using 28-word pairs measured on a 7-point Likert scale. HQ represent the emotional component of the interaction, and PQ embody the behavioral consequences of the interaction. They both independently play a role in the assessment of product attractiveness. Results are presented as median and [minimum; maximum] values.

### Phenomenological approach: UX and opinion on BWS

We applied a phenomenological approach https://www.zotero.org/google-docs/?RnK9O5[39] to understand lived experiences and perceptions of patients and HP. Recruitment ceased after the sample sizes of each group were judged appropriate according to previous related studies [40]. Indeed, Morse et al. noted that six to ten participants could be representative of the population of interest. Semi-structured interviews were conducted between the participant and the interviewer, with specific guides for patients and HP, to describe the individuals’ conceptions of BWS while guaranteeing a common frame for investigation within all participants [41]. Guides are available in S1 Appendix (original language) and S2 Appendix (translated into English).

### Data collection

Interviews with all participants were audiotaped and transcribed. Participants were prompted to answer frankly and describe their feelings and experiences as they live them. Dialogues evolved naturally following the interview guide to ensure that each topic is addressed. We ensured that interactions with participants were free of any preconceived ideas, as detailed by Wojnar and Swanson [42], and, at the beginning of the second round of interviews, we checked the interpretations of claims patients made during the first interviews. After each interview, the participants validated a summary of the interviewer’s understanding of their responses.

The first round of interviews intended to gather the general perspectives of naive participants on BWS (60–90 minutes), and the second round of interviews focused on their experiences with the device to examine the UX (30–60 minutes) [43,44]. The interview of HP (20–60 minutes) was divided into two parts. A fist part allowed a general discussion on BWS before their actual demonstration. The second part involved a more specific analysis of BWS monitoring use.

### Data analysis

We used NVivo 12 software (Qualitative Data Analysis Software from QSR International [45]) to analyze and standardize the data of the participants’ claims to facilitate their interpretation. We applied a four-step approach from the commonly adopted phenomenological method inspired by Giorgi [46,47]:

- Reading of the transcripts to form the first impression (naive understanding);
- Coding of each sentence with NVivo software according to a structural analysis;
- Reviewing of the codes with a comprehensive understanding to look for commonalities and differences following a back and forth movement between the original text and the codes’ categories;
- Disclosure of the descriptions based on the codes’ categories for patients and HP.

Descriptions of results reflect the participants’ most significant perceptions of BWS [48]. One researcher designed the interview guides, interviewed participants, and conducted the qualitative analysis. An external neuropsychologist researcher who selected two interviews with one random patient from each of the three groups cross-checked the codes.

## Results

### Patients

Twenty-two patients were recruited for this study: 7 with BWS a, 8 with BWS b, and 7 with BWS c. Patients’ characteristics are described in Table 1.

**Table 1:** Patients’ characteristics in each group.

Group	BWS a (n = 7)	BWS b (n = 8)	BWS c (n = 7)	All (n = 22)
**Gender ratio** (male/female)	2/5	6/2	1/6	**9/13**
**Age** (years) median [range]	71 [63; 79]	59 [41; 71]	68 [64; 75]	**65,5 [41; 79]**
**Disease duration** (years) median [range]	1 [1; 17]	8,5 [2; 17]	10 [1.5; 14]	**7,5 [1; 17]**
Number of techies*	5	7	5	**17**
**Living alone**	0	2	0	**2**
**Previous knowledge of BWS**	0	2	0	**2**

Techies and non-techies were defined from the patients’ self-description of usual technology mastery (smartphone, computer, *etc*).

#### BWS usability and UX

Each BWS was appraised by seven patients using the SUS and the AttrakDiff questionnaires (one missing data). No correlation was found between age or disease duration and the SUS and AttrakDiff scores (S2 Table).

According to the SUS results, the global usability score for BWS is 87.5 [72.5; 100] (median [range]). The three BWS were assessed as "excellent" to "best imaginable" usability (all scores were above 72.5 on the 100 point scale). SUS scores of BWS a, b, and c were comparable: 90 [77.5; 97.5], 87.5 [72.5; 92.5] and 95 [82.5; 100], respectively.

**Fig 2:**
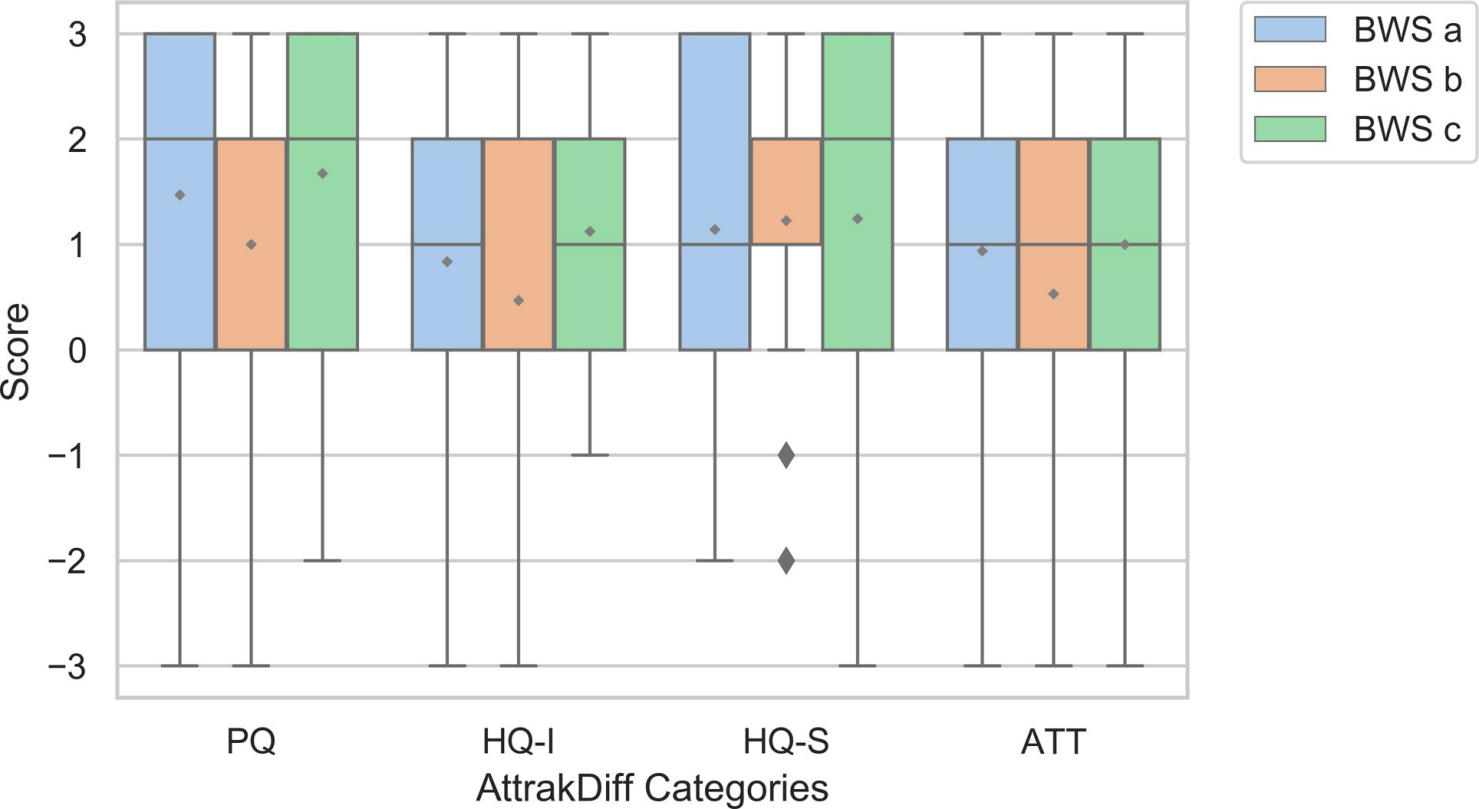
Results of the AttrakDiff questionnaire. Boxplot for each BWS rated with pragmatic qualities (PQ), hedonic qualities linked to the user (identification of the user to the product: HQ-I), hedonic qualities linked to the product (stimulation generated by the product: HQ-S) and attractiveness (ATT).

The four categories of the AttrakDiff questionnaire were assessed positively. Global scores for the three BWS regarding attractiveness, PQ, identification of the user to the BWS and stimulation by the BWS to the user were respectively (median [range]): 1 [-3; 3], 2 [-3; 3], 0 [-3; 3] and 2 [-3; 3]. The rating of the three BWS was positive for 84% of all AttrakDiff items, all categories combined. The three BWS yielded independently very similar results for each AttrakDiff dimension (Fig 2).

#### Phenomenological analysis

According to the two questionnaires and the phenomenological approach analyses, the three groups of patients were equivalent, and the patients’ descriptions allowed us to make general conclusions on BWS monitoring.

*General opinion*. Before this study, 20 out of the 22 patients had not heard of BWS. Finding a new avenue to help patients with PD was the driving force of volunteering, as a majority of patients expressed. The progress inspired by the new term "BWS monitoring" was perceived as positive and the objectives attractive ("Carrying out this type of investigation is very serious."). Patients developed creative thinking about sensors, and many questions emerged from their reasoning linked either to their interests in the device or to device functioning. During the first interview, the expressions used before having seen the BWS reflected positive feelings, interests, direct expectations in the patient’s life, and hopes in potential PD management advances (Table 2). Negative feelings, such as anxiety about handling the device or doubts regarding technological capabilities, were little expressed (Table 2).

**Table 2:** Themes broached across time.

	First Interview		Second Interview
	Before discovery	After discovery	After use
**Positive**	CURIOSITY ENTHUSIASM “NO PROBLEM” EXPECTATIONS HOPES INTEREST	CURIOSITY HOPES ENTHUSIASM EXPECTATIONS INTEREST “NO PROBLEM”	CURIOSITY HOPES EXPECTATIONS ENTHUSIASM GOOD FEEDBACK INTEREST “NO PROBLEM”
**Negative**	CONSTRAINTS ANXIETY DOUBTS NEGATIVE A PRIORI	DISAPPOINTMENT SOCIAL DIFFICULTY DOUBTS ANXIETY CONSTRAINTS NEGATIVE A PRIORI	DISAPPOINTMENT ANXIETY HELP NEEDED CONSTRAINTS DOUBTS INCONVENIENCE / BUG SOCIAL DIFFICULTY

Characters’ sizes reflect the number of patients who expressed those themes during the interviews.

All 22 patients expressed interest in BWS monitoring. The usefulness of the tool triggered expectations and hopes for disease follow-up: "I think that it could replace the diaries which are complicated to fill, I could show that to the doctor instead of the sheets", "it would help manage everyday activities". All patients said the device would not be a problem in their daily life before and after the use ("normal", "trouble-free", "without any issue", the device was "simple", "easy"). The physical characteristics and handling of BWS were trivialized ("aesthetically simple", "no problem"). Patients’ curiosity and enthusiasm increased throughout their discovery (seven patients were enthusiastic before being introduced to the device, 15 before use, and 19 after use).

The patients expressed little anxiety in using the BWS, only two patients manifested apprehension before having seen the device, ten patients after seeing it ("will I be able to use it correctly?", "how should it be done?"), and eight patients after using it. Eleven patients mentioned imagined or lived constraints before use and 12 patients after the one-week use. Few negative a priori conveyed during the first interview: 13 patients had negative preconceptions and four patients considered social embarrassment before use. The device outlook unveiled slight disappointment for four patients and six patients were disillusioned by the device use. Nine patients expressed the wish that the device should not be visible. For example, the ankle bracelet of BWS b regularly reminded people of the electronic parole bracelets. Eight patients pointed out the feeling of being observed or even spied on, wondering about privacy: “We ask ourselves the question, it’s a bit embarrassing, then we move on and we keep on wearing, we forget”, “I removed it to go to the toilet even if I suspected that it was not important for you”. Those patients declared that they did not change their behavior. Six other patients changed their behavior at some point for a short time (extra use of the arm wearing the device, additional time awake, *etc*.). Patients justified the behavior change to provide the necessary information, which was not bothersome considering the possible advantages of a correct monitoring. In rare cases, patients required help with body placement. Finally, despite specific and individual concerns (16 patients expressed slight discomfort or device-related error after use), all patients provided good feedback ("it was a normal week", "I forgot I had it", "everything was fine", "if it helps then we don’t care that it could interfere"). The experiment brought suggestions about design improvement to make the system closer to an everyday object. Indeed, BWS may require specific organization during the day for handling; except for the BWS a that is worn continuously.

After the one-week experiment, 17 patients had a positive, and five had a neutral experience with the BWS use. In addition, 17 patients felt concerned about BWS monitoring other than for this study (S1 Table shows quotations from interviews illustrating those results).

*Suggestions for use*. Suggestions for use were based on the patients’ hopes that BWS monitoring will answer their demands and needs for their PD management. For instance, according to their experiences with the device, patients were able to suggest specific schemes for using the device during their PD follow-up (timing and frequency of monitoring, additional monitoring, extra involvement of the patient, *etc*.) or new ways of using it (other types of measurements, such as cardiac rhythm or other pathologies, for example).

The patients’ enthusiasm increased their interest in attaining a better life with the disease (*i*.*e*., decrease the symptoms). According to 19 patients, clinicians would benefit from patients wearing the devices, as they would be able to adjust the treatment according to the results of BWS monitoring: “if it comes out of the charts, maybe it will allow the doctor to balance the treatment during the day differently. Maybe it will be an effective aid to the doctor”, “it could be useful to me, to adapt the treatment to adapt it more finely than it is”, “during the visit, if we do not remember correctly, I imagine that one can very quickly be mistaken in medication”. Eighteen patients felt the need to know more about their symptoms and 19 expressed the interest to get the results, either to justify the monitoring or their feelings to the physician and then improve the discussion (for 17 patients), or to better understand their disease (for 16 patients): “I did not realize when I had dyskinesia. People had to tell me…I did not see, I did not feel. For example, legs, arms, I did not realize. It helps people to realize. And it seems that there are also people who do not differentiate between symptoms and dyskinesia; for these people, it can be useful too”. For 18 patients, the general exchange during consultations was incomplete because they could not have the time to discuss everything they wanted. In parallel, respectively eleven and nine patients were not satisfied with their treatment and PD follow-up at the time of the study. Finally, twelve patients also thought about research (“we could compare the benefits of different treatments, for example”) and the advantages of BWS monitoring for PD knowledge (“when we understand what is happening, we can act”).

### Health professionals

The nine HP were working part-time or full time in a movement disorder expert center. The three nurses and three of the six clinicians were women. Seven HP knew about BWS monitoring before the interview. Only two of those were aware of other BWS than the BWS a.

#### Phenomenological analysis

As none of the HP had used BWS monitoring before the study, the discussion focused on the practice. The presentation of the three BWS at mid-interview provided a general overview of the existing systems. HP’s perceptions of each BWS and the different resulting reports, as well as device-specific suggestions of improvements, were not the purpose of this study so the information is not detailed here.

*General opinion*. A significant interest in BWS monitoring was highlighted by all HP. At the end of the interview, all HP had expressed enthusiasm to use this new technology for PD, showing positive and negative perceptions that were balanced. The attraction was driven by a conceivable beneficial evolution of medicine (“I think it’s more reliable than an interrogation”, “I think it’s better than the diary they have to fill out. It’s difficult.”). On the contrary, 6 HP expressed some reluctance to use it in specific conditions. They expressed concerns about the role of technology in a human-to-human relationship that characterizes medical care (“The disadvantages…more ethically, it replaces the patient-doctor relationship. We say that we are going to do telemedicine and tell the patient ‘you no longer come for a consultation, you will send me your curves and I tell you how to adapt your treatments‴). The number of references to “trust” in relation to “doubts” regarding the technology was equal. The thoughts on BWS monitoring revealed questions about the functioning of devices (9 HP) and the need to help them use such a device in real-life (8 HP out of 9). HP required that particular attention had to be paid to their training (issues of how, when and for which patient BWS monitoring should be used, as well as how to apply the results) and to the logistical support (set up the monitoring and instruct the patients, give/return the device, get the results, *etc*). Finally, HP expressed the need of scientific proof of BWS usefulness to improve patients’ PD. All HP suggested different ways to take advantage of motor symptoms measurement.

*Suggestions for use*. All HP expressed ideas for clinical care, and 5 HP also mentioned research purposes. The treatment adaptation was the main suggestion for using BWS in care. All HP considered BWS as a possible support tool to adjust oral medication, pump delivery, or deep brain stimulation, or to decide on the transition to advanced therapies. Three HP suggested that it could help minimizing or avoiding visits or hospitalizations. Another suggestion (3 HP) was to use BWS monitoring results for the therapeutic education of patients. Regarding research, HP thought the development of new-targeted treatments would be eased by motor symptoms measurement, and they suggested focusing on the study of other movement disorders. Further, 3 HP suggested the use of BWS monitoring to create an automatic system for real-time treatment adjustments according to motor symptoms measurement, and two contemplated direct feedback from the measure to an actuator, such as auditory cueing or fall alarms.

## Discussion

This study provided insights into the patients’ and HPs’ experiences of BWS. Both demonstrated a great interest in this new application, and both advantages and disadvantages of using the devices were proposed. To our knowledge, this is the first study that allowed confrontation of viewpoints of the two stakeholders on several PD specific devices.

Validated questionnaires revealed that the three BWS were acceptable (good usability assessed with the SUS) [36] and attractive (positive UX results with the AttrakDiff). Still, the ratings show there was room for improvement. Disparities were observed between the three patients’ groups (especially regarding the disease duration and the gender ratio—Table 1), and the number of patients in each group was limited, but no correlation was found between the patients’ characteristics and the questionnaires scores (S2 Table).

To summarize the participants’ perception, and on the one hand, patients were enthusiastic and engaged in the experience because they hoped that a new tool could improve their PD management. The device use also raised expectations as the handling was largely felt simple and practical, and the device considered as an everyday life object. The patients expressed little worries about the potential constraints of their use and dissatisfaction, which did not have a strong effect on the general perception of BWS. Instead, the patients were curious and proposed improvements in device performance in terms of simplifying the manipulation (especially for patients living alone—Table 1) or using new materials. On the other hand, HP expressed some concerns about the implementation of BWS monitoring into their practice mainly because of the device logistics. Support from the expert staff was required, and time dedicated to training on the interpretation and reviewing the results was the limiting factor. However, they appreciated the accuracy and reliability that a continuous and objective ambulatory assessment of motor symptoms with high resolution could provide. HP also appreciated the ease with which the results could be read, as well as the ease with which patients could wear these devices.

The Movement Disorder Society (MDS) supports the implementation of BWS monitoring in clinical care and research [23]. This task force published a roadmap to facilitate monitoring adoption, given the technology’s maturity compared to the slow progression of its use in clinical care and research. Still, in this study, HP voiced fundamental requirements before complete adoption. Both patients and HP shared enthusiasm and suggested ways to use BWS monitoring in treatment adaptations or research advances. However, HP formulated the need for support measures for its implementation in practice that may not yet be totally deployed by healthcare organizations. HP also still needed an extended proof of the clinical relevance. Indeed, the technology readiness faces a lack of clinical evidence in its input for PD symptoms’ improvement, which may impact the willingness of HP in using such systems. While Santiago et al. [49] demonstrated that physicians might already use the technology to facilitate treatment adaptation, the technology has not yet been adopted globally. Patients may be more willing to use BWS compared to HP. Patients indicated that they are ready to include BWS in their care pathway. This study is consistent with others that have shown very good acceptance of and readiness to wear quite complex systems [50,51]. According to Ozanne, “the benefits were valued higher than the possible inconvenience of wearing the sensors” (page 6) [52].

Thus, even though previous studies endorsed the use of BWS monitoring for PD in clinical practice and research [22], gaps in the application that restrain the full deployment have been identified. Healthcare organizations should develop a support system required by HP to follow MDS guidance and facilitate BWS monitoring while managing the barriers to BWS use [52]. This study offers strong support that BWS monitoring may be a support for clinicians in adjusting the treatment. Both patients and HP shared the idea that BWS monitoring could also facilitate discussion and benefit the interactions more than using the symptoms diary, as in Fisher’s study [53]. BWS could help instruct patients about their symptoms and assist therapeutic education.

Moreover, patients might feel integrated into their care management when receiving feedback on their assessment with BWS [50], which would increase treatment adherence [54]. The active involvement of patients in their care through BWS monitoring might improve their situation and increase their quality of life and/or wellbeing [55]. Besides, this study showed that a few patients adapted their behavior without difficulty in order to improve the monitored movements and then get encouraged by the results. The motivation generated by the possible consequences of BWS monitoring on health might encourage patients to pay close attention to their condition, so they adopt actions that may be more appropriate, which can be observed for fall prevention awareness, for example [56]. Further investigation should be done to explore whether BWS can generate a placebo effect that would enhance the patient’s situation, as PD is known to be easily influenced by placebos [57]. That also gives rise to the need for controlled clinical studies when involving BWS monitoring. Furthermore, HP broached the opportunity to create automatic treatment delivery systems based on the results of the objective monitoring of BWS. Experts and manufacturers should explore the chances to set up such a system in compliance with the technical and the users’ perspectives.

### Methodological considerations

The strength of this study is that it considered the viewpoints of both patients and HP on three different BWS for PD before and after their use. The reliability of data is increased by the absence of inter-researcher bias, as only one researcher conducted the interviews, transcribed them, and analyzed the transcripts. Moreover, the external researcher who performed cross-checking of the codes agreed with the codes’ categories and their description. During the second interview, patients confirmed the findings that emerged from the first interview, which decreased the conformity bias. On the one hand, a selection bias was one limitation since it was not possible to conduct the one-week experiment with the BWS b when patients could not guarantee internet connection. Maybe non-technophile patients would have expressed more negative feedback after complicated handling (number of techies in Table 1).

Moreover, the diversity of patients’ profiles might have shaped their perception of the BWS tested. Additionally, the UX may have been affected by the seasonality effect, as all interviews of patients were performed during winter and fall. Some perceptions of BWS might have been different if the interviews took place during the summer months (for instance, greater visibility of the device to others, bands that keep warm and cause sweating). On the other hand, the phenomenological approach adapted from van Manen [58] is a well-established method for qualitative research and guarantees the trustworthiness [59] of this study’s interpretation.

The findings that emerged from this study are hypothesis-generating rather than definitive; no consensus is aimed to be pronounced in this study. Perhaps, in a standard care context, those same patients would not have as many comments and suggestions of use. Therefore, the current study could be reproduced in other movement disorder centers to collect different viewpoints from various PD populations and help personalize BWS use to each one.

## Conclusion

The study of the patients’ and HP’s perceptions of BWS monitoring seems to favor its use in research and care practice. The UX of patients was positive for the majority of patients, supporting the very good usability and the attractiveness of these study’s devices. Patients and HP offered complementary opinions and suggestions for use. Both sides expressed their interest and enthusiasm in BWS monitoring. Few developments of new devices involving human-machine interaction consider the UX early in the process, but patients’ and professionals’ perceptions may help develop a system that will respond to the needs and the expectations of all stakeholders. Adhering to the recommendations of patients and professionals would maintain a final optimal use of BWS monitoring and its correct implementation to standards of care [60]. Although, clear clinical evidence of the relevance for PD care still remains necessary.

This study draws reliable information on the implementation of BWS in real-life situations in clinical practice and the real lives of PD patients. From those outcomes, healthcare organizations can carry out actions to answer the needs of patients and HP to organize an effective and efficient adoption of BWS monitoring. Manufacturers can also make the most of this experience to help organizations seek improvements in PD care through BWS monitoring.

## Supporting information

S1 Table Quotations from interviews, which illustrate the general perception of BWS of patients. (DOCX)

S2 Table Association study between the questionnaires scores and patients’ characteristics. (DOCX)

S1 Appendix (PDF)

S2 Appendix (PDF)

## Abbreviations

ATT: Attractiveness
BWS: Body-Worn Sensors
HP: Health Professionals
HQ: Hedonic Qualities
HQ-I: Hedonic Qualities—Identification
HQ-S: Hedonic Qualities—Stimulation
PD: Parkinson’s disease
PQ: Pragmatic Qualities
SUS: System Usability Scale
UX: User Experience
