# Supplementary material for: Body-Worn Sensors for Parkinson’s disease: A qualitative approach with patients and healthcare professionals
Source: PLoS One. 2022 May 5;17(5):e0265438. doi: 10.1371/journal.pone.0265438 (PMC9070870; doi:10.1371/journal.pone.0265438)
Supplement: S1 Table — (DOCX) [file pone.0265438.s001.docx]

**S1 Table: Quotations from interviews, which illustrate the general perception of BWS of patients.**

|  | **Before use** | **After use** |
| --- | --- | --- |
| **Hopes** | *01UR: “maybe it's going to come out of extraordinary stuff”*  *06UR: “It's curiosity [...] we want to understand”*  *12UR: “It's great because I never know where to put it on the sheets. It's always very complicated, it's better if there is a device that records them. It's an advantage. It will replace the sheet which is very difficult to fill”*  *15UR: “I think it can be an aid with several drawers. I feel like it can be useful to patients and doctors, and to any team around the patient”*  *30UR: “an impression like that, I say to myself: “finally”, this is my point of view, I say finally because from the start I have no reference bar”* | *01UR: “Anything that can improve my life, I'm up for it”*  *08UR: “it would be an assistance, a more precise additional measure”*  *11UR: “I see as a useful aid to doctors in relation to the objective sign which is interesting, in relation to what the patients can bring back from their experiences which are subjective and possibly more romanticized...which doesn't have the rigor of a recording like that”*  *12UR: “It helps the patients to tell, it helps the relationship between the patients and the neurologists. To put it exactly like that, it's that neurologists can tailor treatments to the things that happen, to the lives of the patients”* |
| **Expectations** | *09UR: “So it will be much more precise and useful than if I wrote down the moments of difficulty hour by hour. It will be more precise and more reliable”*  *14UR: “I manage to describe how I feel in consultation a little bit but not so much, we are always a little bit...vague. I could surely say more things during the consultation”*  *30UR: “It would first be useful for the practitioner because when he asks us questions, we have answers...well, I’m too neutral in my dialogues, I don’t know how to describe in fine detail if things are better, if it doesn't get better at such a time of the day, 6 months later. I can't say if it gets better than such 6 months before, at some other time of the day, it's subjective.”* | *09UR: “I give a vague answer but it is not objective. While with these measures, we can see an objective basis to inform the doctor”*  *11UR: “In my idea it was a device that was going to be more than a clinician”*  *14UR: “actually trying to see, trying to clarify a little, the why of these movements, these tremors which are sometimes increased and sometimes not. Why are there days when everything is going well and days when it is not going well because it is true that indeed we do not always see causes”*  *30UR: "Finally we are going to measure something at home because for 7 years, nobody has measured anything at all, I do not know where I am compared to what I was and finally something concrete"* |
| **Fears** | *01UR: "in front of people it's complicated"*  *10UR: “I was a bit scared, I thought I was going to carry a lot of things on me”*  *14UR: ”I am not saying that it is not good but I think that one should not make a blind confidence. We must also analyze what we feel what we see. You can't just rely on the device”*  *27UR: “Sometimes there are things we can have that we prefer not to know”* | *01UR: “Can we check if it worked well? It’s the fear of not using it well”*  *03UR: “On the other hand, there was a moment when I was afraid that it would go away because of my dyskinesias. It can't go, does it hold up well?”*  *04UR: “I felt a little spied on”*  *06UR: “it is an apprehension compared to the evaluation of the stage of my disease”*  *31UR: “there are people who will not be able to bear to see evolution”* |
| **Questions** | *02UR: “Can we visualize the results? How is it transcribed to you?”*  *15UR: “Do you compare the results of different patients with each other too? Will having this device on me make me more vigilant for example?”*  *29UR: how do you know it's related to the disease or not?”* | *06UR: “I was curious, I said to myself but what does it record in fact? Is it the movement of the wrist, is it the whole shoulder... Is it the rhythm... I wondered what evaluations it gave”*  *09UR: “Can we make the recordings speak? Can we watch a recording to understand what it is?”* |

Quotations were translated from French, the interpretation sense may slightly differ from one language to another.
