# Supplementary material for: Body-Worn Sensors for Parkinson’s disease: A qualitative approach with patients and healthcare professionals
Source: PLoS One. 2022 May 5;17(5):e0265438. doi: 10.1371/journal.pone.0265438 (PMC9070870; doi:10.1371/journal.pone.0265438)
Supplement: S2 Table — (DOCX) [file pone.0265438.s002.docx]

**S2 Table: Association study between the questionnaires scores and patients’ characteristics**

|  | **SUS** | | **AttrakDiff total score** | | **ATT** | | **PQ** | | **HQ-I** | | **HQ-S** | |
| --- | --- | --- | --- | --- | --- | --- | --- | --- | --- | --- | --- | --- |
|  | ***r*** | ***p*** | ***r*** | ***p*** | ***r*** | ***p*** | ***r*** | ***p*** | ***r*** | ***p*** | ***r*** | ***p*** |
| **Age** | 0.18 | 0.44 | -0.03 | 0.89 | -0.07 | 0.75 | 0.12 | 0.60 | 0.16 | 0.50 | -0.21 | 0.35 |
| **Disease Duration** | -0.09 | 0.70 | -0.08 | 0.72 | -0.289 | 0.20 | 0.03 | 0.91 | 0.10 | 0.65 | -0.09 | 0.69 |

Pearson correlation coefficients (r) with p-values (p).
