# Supplementary material for: Body-Worn Sensors for Parkinson’s disease: A qualitative approach with patients and healthcare professionals
Source: PLoS One. 2022 May 5;17(5):e0265438. doi: 10.1371/journal.pone.0265438 (PMC9070870; doi:10.1371/journal.pone.0265438)
Supplement: S1 Appendix — (PDF) [file pone.0265438.s003.pdf]

1. Guide d'entretien patients

1.1. Premier entretien

1.1.1. Introduction (10 minutes)

- Explication du déroulé de l'étude : 3 phases
  - 1er entretien aujourd'hui
  - expérience de l'utilisation d'un actimètre à domicile pendant une semaine
  - 2ème entretien dans une semaine
- Aujourd'hui : entretien, description du dispositif pour l'utilisation à domicile et prise en main par le patient, signature consentement de participation
- Avez-vous déjà entendu parler de l'actimétrie ?
  Si oui : quel est votre définition ?
  Si non : pouvez-vous deviner ?
- Environ 1h d'entretien sur différents sujets :
  - questions sur le patient et sur son mode de vie avec la MP aujourd'hui (introduction)
  - questions sur ce qui a été compris de l'actimétrie et ce qui est attendu de ce type de dispositif (thème #1)
  - présentation du fonctionnement de l'actimètre pour l'expérience à domicile
  - questions sur le point de vue sur cette technologie et les objectifs de l'actimétrie pour le suivi de la maladie de Parkinson (thèmes #2 et #3)
- Pas de bonne ou de mauvaise réponse, pas de jugement : ce n'est pas une évaluation, intérêt dans l'opinion personnel, le patient peut être complètement franc
- Enregistrement de la conversation pour pouvoir retranscrire correctement ce qui est dit pendant l'entretien et pouvoir échanger de manière plus libre : cet enregistrement ne sera utilisé que dans le cadre de cette étude et toutes les informations recueillies resteront anonymes.
- Le patient est libre de vouloir arrêter l'enregistrement ou ne pas répondre à une question, le patient peut poser des questions à n'importe quel moment

1.1.2. Questions

Introduction générale (10 minutes)

1. Pouvez-vous vous présenter ?
2. Quel est / était votre métier ? / Quelles sont vos activités ?
3. Pouvez-vous décrire une de vos journées typiques ?
4. Avez-vous une habitude quotidienne ? Quelque chose que vous faites tous les jours ?
5. Pouvez-vous décrire l'historique de votre maladie rapidement ?
6. Diriez-vous que vous êtes satisfait(e) de votre traitement ?
7. Pouvez-vous donner votre avis sur votre suivi médical de la maladie ?
8. Comment êtes-vous pris en charge pour la maladie de Parkinson ? / Que faites-vous en particulier pour votre maladie de Parkinson ?
9. Souhaitez-vous connaître davantage sur vos symptômes ?
10. Qu'aimeriez-vous changer pour améliorer votre vie avec la maladie ?

Thème #1 : Compréhension et attentes de l'actimétrie (5 minutes)

1. Que pensez-vous de l'utilisation de nouvelles technologies pour suivre votre maladie ?
2. Comment décririez-vous l'intérêt que vous portez pour les nouvelles technologies ?
3. Quelle est la place de ce type d'outil dans votre vie de tous les jours ?
4. Utilisez-vous des outils technologiques pour votre maladie de Parkinson ? Lesquels ?
5. Est-ce la première fois qu'on vous présente l'actimétrie ?
   a. Quelle a été votre première réaction face à la présentation de l'actimétrie ?
   b. Quel est le premier mot qui vous vient à l'esprit pour parler ou décrire ces dispositifs ? Diriez-vous que c'est positif ou bien négatif ? Pourquoi ?

Présentation du dispositif destiné au patient (5 minutes)

Thème #2 : Perception du suivi par l'actimétrie (10 minutes)

1. Que vous inspire cet appareil ? Comment réagissez-vous à ce capteur ?
2. Qu'est-ce qui vous a le plus impressionné dans cette présentation d'actimètre ? De quoi vous rappelez-vous ?
3. Dans quelle mesure pensez-vous que cela vous sera utile ?
4. Pouvez-vous donner des exemples de situations qui, selon vous, pourraient bénéficier des actimètres ?
5. Pouvez-vous donner des exemples de cas où les actimètres pourraient ne pas être un bon outil de surveillance de la MP ?
6. Selon vous, en quoi le port d'un capteur sur votre corps pour enregistrer vos symptômes serait-il un avantage/un inconvénient ?
7. Pensez-vous à des fonctionnalités que vous aimeriez voir avec les outils de suivi de la MP ?
8. Comment vivez-vous le fait de récupérer un actimètre pour suivre votre maladie pendant une semaine ? Et de devoir porter l'appareil sur votre corps tous les jours après notre entretien ?
9. Avez-vous quelque chose à ajouter ?

Thème #3 : Attentes et perspectives (10 minutes)

1. Quelles sont vos attentes sur cette semaine d'enregistrement avec un dispositif d'actimétrie ?
2. Quel est votre opinion sur le suivi de la maladie grâce à des technologies tels que les actimètres ?
3. Diriez-vous que vous êtes intéressés par les résultats d'enregistrement ? Seriez-vous attiré par la compréhension des mesures effectuées par ces dispositifs ? Pourquoi ?
4. Quel est l'impact que vous ressentez de l'actimétrie sur la gestion des symptômes moteurs ?
5. Quelle valeur voyez-vous en l'actimétrie ?
6. Croyez-vous que l'utilisation de l'actimétrie pourrait vous concerner en dehors de cette étude ?
   a. Pensez-vous que suivre l'évolution de votre maladie avec ce type de dispositifs pourra améliorer vos conditions de vie avec la maladie ?
   b. Quelle serait d'après vous l'utilisation que vous en feriez si vous possédiez ce dispositif ?
7. Quelles sont vos attentes sur l'actimétrie de façon générale ?
8. Avez-vous d'autre idées à ajouter ? D'autres remarques ?

1.1.3. Conclusion (15 minutes)

- Rappel des prochaines étapes : expérimentation à domicile pendant une semaine puis 2ème entretien
- Description du fonctionnement de l'actimètre avec lecture du manuel d'utilisation
- Mise en place de l'actimètre

1.2. Deuxième entretien

1.2.1. Introduction (10 minutes)

- Rappel du déroulé de l'étude : 3 phases
  - 1er entretien il y a une semaine
  - expérience de l'utilisation d'un actimètre à domicile pendant une semaine
  - 2ème entretien aujourd'hui
- Aujourd'hui : entretien, questionnaires, signature consentement de participation
- Environ 1h d'entretien sur différents sujets :
  - questions sur le patient et sur son mode de vie avec la MP aujourd'hui (introduction)
  - questions sur ce qui a été retenu de l'expérience avec le dispositif (thème #1 et #2)
  - questions sur les idées et les objectifs de l'actimétrie pour le suivi de la MP (thème #3)
- Pas de bonne ou de mauvaise réponse, pas de jugement : ce n'est pas une évaluation, intérêt dans l'opinion personnel, le patient peut être complètement franc

- Enregistrement de la conversation pour pouvoir retranscrire correctement ce qui est dit pendant l'entretien et pouvoir échanger de manière plus libre : cet enregistrement ne sera utilisé que dans le cadre de cette étude et toutes les informations recueillies resteront anonymes.
- Le patient est libre de vouloir arrêter l'enregistrement ou ne pas répondre à une question, le patient peut poser des questions à n'importe quel moment

1.2.2.Questions

<u>Introduction générale (5 minutes)</u>

1. Avez-vous changé vos habitudes par rapport à la maladie depuis le premier entretien ?
2. Comment s'est déroulé la semaine par rapport à vos symptômes ?
3. Pensez-vous à quelque chose que vous voudriez ajouter par rapport au premier entretien ?

<u>Thème #1 : Retour d'utilisation d'un dispositif d'actimétrie (10 minutes)</u>

1. Comment décririez-vous votre semaine avec le dispositif ?
   Quel est le premier mot qui vous vient à l'esprit pour décrire cette semaine d'utilisation ?
2. Votre première expérience avec le dispositif a-t-elle été positive ou négative ? Pouvez-vous expliquer pourquoi ?
3. Qu'est-ce qui vous a encouragé à continuer à utiliser le dispositif ?
   Qu'est-ce qui vous a fait arrêter d'utiliser le dispositif ?
4. Quel est votre point de vue sur le design / l'ergonomie du dispositif ?
5. Comment avez-vous vécu l'utilisation et le port du dispositif tous les jours ?
6. Le dispositif vous a-t-il gêné ou a-t-il été inconfortable à des moments précis / tout le temps ?
   Pouvez-vous donner des cas concrets ?
7. Avez-vous vécu le port / l'utilisation du dispositif comme une contrainte ?
   Quelles ont été ces contraintes liées au dispositif dans la vie de tous les jours ? A des moments particuliers ?

<u>Thème #2 : Perception du suivi par l'actimétrie (10 minutes)</u>

1. Est-ce que vous en avez parlé autour de vous ?
   a. Si oui, comment avez-vous présenté le dispositif ? Quels ont été les remarques ? Et comment avez-vous pris ces remarques ?
   b. Si non, avez-vous reçu des commentaires sans que vous en parliez ? Est-ce que certaines personnes ont remarqué que vous portiez un dispositif ? Cela vous a-t-il gêné que le dispositif soit visible ?
2. Avez-vous perçu des changements dans votre comportement à cause du port du dispositif, dans l'accomplissement de vos activités quotidiennes ?
3. Que pensez-vous du fait que les mouvements que vous avez effectués à un moment donné soient enregistrés ?
4. Pensez-vous à autre chose que vous aimeriez partager sur cette expérience du dispositif ?

<u>Thème #3 : Attentes et perspectives (5 minutes)</u>

1. Quelles sont vos attentes de l'actimétrie maintenant que vous avez utilisé le dispositif ?
2. Quel est votre opinion sur le suivi de la maladie grâce à des technologies tels que les actimètres ?
3. Quel est l'impact que vous ressentez de l'actimétrie sur la gestion des symptômes moteurs ?
4. Quelle valeur voyez-vous en l'actimétrie dorénavant ?
5. Croyez-vous que l'utilisation de l'actimétrie pourrait vous concerner en dehors de cette étude ?
   a. Pensez-vous que suivre l'évolution de votre maladie avec ce type de dispositifs pourra améliorer vos conditions de vie avec la maladie ?
   b. Quelle serait d'après vous l'utilisation que vous en feriez si vous possédiez ce dispositif ?
6. En un mot, quelle est votre perception de l'actimétrie de façon générale ?
7. Pensez-vous à des caractéristiques du système que vous souhaitez améliorer / changer / ajouter ?
8. Si ce n'est pas ce dispositif, quel serait d'après vous l'outil idéal pour mesurer les symptômes moteurs de la MP ?
9. Avez-vous d'autre idées à ajouter ? D'autres remarques ?

1.2.3. Questionnaires (10 minutes)

- Test de l'utilisabilité : SUS
- Test de l'expérience utilisateur : AttrakDiff

1.2.4. Conclusion (5 minutes)

2. Guide d'entretien professionnels de santé

2.1. Introduction (10 minutes)

- Environ 1h d'entretien sur différents sujets :
  - questions sur la pratique clinique et comment les symptômes de la MP sont évalués dans la pratique (introduction)
  - questions sur ce qui est connu de l'actimétrie et perception (thèmes #1 et #2)
  - questions sur les attentes vis-à-vis de la technologie (thème #3)
- Pas de bonne ou de mauvaise réponse, pas de jugement : ce n'est pas une évaluation, intérêt dans l'opinion personnel, le participant peut être complètement franc
- Enregistrement de la conversation pour pouvoir retranscrire correctement ce qui est dit pendant l'entretien et pouvoir échanger de manière plus libre : cet enregistrement ne sera utilisé que dans le cadre de cette étude et toutes les informations recueillies resteront anonymes.
- Le participant est libre de vouloir arrêter l'enregistrement ou ne pas répondre à une question, le participant peut poser des questions à n'importe quel moment

2.2. Questions

<u>Introduction générale (5 minutes)</u>

1. Diriez-vous que vous êtes satisfait des moyens techniques dont vous disposez pour le suivi des patients dans leur MP ?
2. Pouvez-vous donner votre avis sur les modalités de suivi de la MP ?
3. Que souhaiteriez-vous changer pour améliorer le suivi de vos patients ?
4. Comment décririez-vous votre pratique des nouvelles technologies ? Quelle est la place de ce type d'outil dans votre pratique clinique ?

<u>Thème #1 : Compréhension et attentes (5 minutes)</u>

1. Que pensez-vous de l'utilisation des nouvelles technologies pour suivre l'évolution des maladies chroniques ?
2. Avez-vous déjà entendu parler de l'actimétrie ?
   a. Si oui : quelle est votre définition ?
   b. Sinon : pouvez-vous imaginer ?
3. Est-ce la première fois que vous participez à un projet avec des actimètres ?
   a. Si oui, quelle est votre réaction ?
   b. Si non, quelle a été votre première impression ?
4. Avez-vous changé votre perception sur le suivi par actimétrie ? Qu'en est-il aujourd'hui ?

<u>Présentation des 3 systèmes et des rapports (5 minutes)</u>

<u>Thème #2 : Perception du suivi par l'actimétrie (10 minutes)</u>

1. Quelle est votre opinion sur le suivi de la MP à l'aide de technologies telles que les actimètres ?
2. Dans quelle mesure pensez-vous que l'actimétrie vous serait actuellement utile ?
3. Pouvez-vous donner quelques exemples de situations qui, selon vous, pourraient bénéficier de l'actimétrie ?
4. Pouvez-vous donner des exemples de cas où l'actimétrie pourrait ne pas être un bon outil pour le suivi de la MP ?

5. À votre avis, faire porter un actimètre à un patient serait-il :
    a. un avantage pour votre pratique clinique et/ou pour le patient ?
    b. un inconvénient pour votre pratique et/ou pour le patient ?
6. Pensez-vous à des fonctionnalités que vous aimeriez voir avec les actimètres ?
7. Avez-vous quelque chose à ajouter ?

<u>Thème #3 : Attentes et perspectives (10 minutes)</u>

1. Quelles sont vos attentes concernant l'actimétrie ?
2. Quelle valeur voyez-vous dans l'actimétrie ?
3. Voyez-vous une utilisation plutôt orientée vers la pratique courante ? Pouvez-vous me donner des cas concrets ?
4. Ou de la recherche ? Avez-vous des exemples à donner ?
5. Quel impact présentez-vous sur la gestion des symptômes moteurs ?
6. Pensez-vous que l'utilisation de l'actiémtrie pourrait vous concerner ?
7. Pensez-vous que suivre l'évolution de vos patients avec ce type d'appareil améliorera vos conditions de travail ? Si oui, comment ? Si non pourquoi ?
8. Comment utiliseriez-vous cet appareil pour votre pratique si vous le possédiez ?
9. Avez-vous d'autres idées à ajouter ? D'autres commentaires ?

2.3. Conclusion (5 minutes)
