# Supplementary material for: Body-Worn Sensors for Parkinson’s disease: A qualitative approach with patients and healthcare professionals
Source: PLoS One. 2022 May 5;17(5):e0265438. doi: 10.1371/journal.pone.0265438 (PMC9070870; doi:10.1371/journal.pone.0265438)
Supplement: S2 Appendix — (PDF) [file pone.0265438.s004.pdf]

Interview guides translated from French into English

1. Patient's interview guide

1.1. First interview

1.1.1. Introduction (10 minutes)

- Explanation of the course of the study: 3 phases
    - 1st interview today
    - experience of using a BWS at home for a week
    - 2nd interview in a week
- Today: interview, BWS device presentation for the home experiment and patient's handling, study participation consent

Have you ever heard of body-worn sensors/monitoring?

If yes: what is your definition?

If not: can you guess?

- About 1 hour of interview on different topics:
    - questions about the patient and his lifestyle with PD today (introduction)
    - questions on what has been understood from BWS monitoring and what is expected from this type of device (topic #1)
    - presentation of the BWS that will be taken for the home experiment
    - questions on the point of view on this technology and the objectives of BWS for monitoring PD (topics #2 and #3)
- No right or wrong answer, no judgement: this is not an assessment, interest in personal opinion, the participant can be completely frank
- Recording of the conversation to be able to correctly transcribe what is said during the interview and to be able to exchange more freely: this recording will only be used within the purpose of this study and the information collected will remain anonymous.
- The participant is free to want to stop the recording or not answer a question, the participant can ask questions at any time

1.1.2. Questions

<u>General introduction (10 minutes)</u>

1. Can you introduce yourself?
2. What is / was your job? / What are your activities?
3. Can you describe one of your typical days?
4. Do you have a daily habit? Something you do every day?
5. Can you briefly describe the history of your illness?
6. Would you say that you are satisfied with your treatment?
7. Can you give your opinion on your medical follow-up of the disease?
8. How is your PD managed? / What do you do in particular for your PD?
9. Would you be interested in learning more about your symptoms?
10. What would you like to change to improve your life with the disease?

<u>Topic #1: Understanding and expectations (5 minutes)</u>

1. What do you think of using new technologies to track your disease?
2. Do you use technological tools for your PD? Which ones?
3. How would you describe your interest in new technologies?
4. What is the place of this type of tool in your everyday life?
5. Is this the first time that sensors are presented to you?
    a. What was your first reaction to the presentation of sensors?
    b. What is the first word that comes to mind to speak or describe these devices? Would you say it is positive or negative? Why?

<u>Device presentation (5 minutes)</u>

<u>Topic #2: Perception of the follow-up by monitoring with sensors (10 minutes)</u>

1. What does this device inspire you? How do you react to this sensor?
2. What impressed you most about this presentation on BWS monitoring? What do you remember?

3. To what extent do you think this will be useful to you?
4. Can you give examples of situations that you think could benefit from monitoring with sensors?
5. Can you give examples of cases where monitoring with sensors might not be a good tool for monitoring PD?
6. How do you think wearing a sensor on your body to record your symptoms will be an advantage/disadvantage?
7. Do you think of features you would like to see with disease monitoring tools?
8. How do you live with getting a tool to track your illness for a week? And having to wear the device on your body every day after our interview?
9. Do you have anything to add?

Topic #3: Expectations and perspectives (10 minutes)

1. What are your expectations for this week of recording with such a device?
2. What is your opinion on monitoring the disease using technologies such as sensors?
3. Would you say that you are interested in the results of the monitoring? Would you be interested in understanding the reports obtained? Why?
4. What impact do you feel from sensor monitoring on managing motor symptoms?
5. What value do you see in sensor monitoring?
6. Do you think that the use of sensors could concern you outside of this study?
    a. Do you think that following the evolution of your disease with these types of devices can improve your living conditions with the disease?
    b. What do you think you would use it for if you had this device?
7. What are your expectations for BWS monitoring in general?
8. Do you have any other ideas to add? Any other comments?

1.1.3. Outro (15 minutes)

- Reminder of the next steps: experimentation at home for a week then 2nd interview
- Description of the operation of the BWS with reading of the user manual
- Installation of the BWS

1.2. Second interview

1.2.1. Introduction (10 minutes)

- Reminder of the course of the study: 3 phases
    - 1st interview a week ago
    - experience of using a BWS at home for a week
    - 2nd interview today
- Today: interview, questionnaires, signature consent to participate
- About 1 hour of interview on different topics:
    - questions about the patient and his lifestyle with PD today (introduction)
    - questions on what was learned from the experience with the device (topics #1 and #2)
    - questions on the ideas and objectives of BWS for monitoring PD (topic #3)
- No right or wrong answer, no judgement: this is not an assessment, interest in personal opinion, the participant can be completely frank
- Recording of the conversation to be able to correctly transcribe what is said during the interview and to be able to exchange more freely: this recording will only be used within the purpose of this study and the information collected will remain anonymous.
- The participant is free to want to stop the recording or not answer a question, the participant can ask questions at any time

1.2.2. Questions

General introduction (5 minutes)

1. Have you changed your habits about the disease since the first interview?
2. How did the week go about your symptoms, fatigue, etc.?
3. Do you have anything else you would like to add regarding our first interview?

Topic #1: Feedback on the use of a sensor for one week (10 minutes)

1. How would you describe your week with the device? / What is the first word that comes to mind to describe this week?

2. Was your first experience with the device positive or negative? Can you explain why?
3. What encouraged you to continue using the device? / What made you stop using the device?
4. What is your point of view on the design / ergonomics of the device?
5. How did you experience the handling and installation / removal of the device every day?
6. Did the device bother you or was it uncomfortable at specific times / all the time? Can you give concrete examples?
7. Did you experience wearing / handling the device as a constraint? Was wearing the device all day complicated? What were these constraints linked to the device in everyday life? At particular times?

Theme #2: Perception of the follow-up with sensors (10 minutes)

1. Have you talked about the monitoring with sensors around you?
   a. If yes, how did you present the device? What were the comments? And how did you take these remarks?
   b. If not, have you received comments without your mentioning them? Have some people noticed that you are wearing a device? Did you mind that the device was visible?
2. Have you noticed any changes in your behavior due to the wearing of the device, in the performance of your daily activities?
3. What do you think about the fact that the movements that you performed are monitored?
4. Do you think of anything else you would like to share about this device experience?

Theme #3: Expectations and perspectives (5 minutes)

1. What are your expectations from monitoring now that you have used the device?
2. What is your opinion on monitoring the disease using technologies such as sensors?
3. What impact do you feel from the monitoring on managing motor symptoms?
4. What value do you see in monitoring with sensors now?
5. Do you think that the monitoring with sensors could concern you outside of this study?
   a. Do you think that following the evolution of your disease with these types of devices can improve your living conditions with the disease?
   b. If you had this device, what use do you think you would have of it?
6. In one word, what is your perception of the monitoring with sensors in general?
7. Do you think of features of the system that you want to improve / change / add?
8. If not this device, what do you think would be the ideal tool to measure the motor symptoms of PD?
9. Do you have any other ideas to add? Any other comments?

1.2.3. Questionaires (10 minutes)

- Test of the usability: SUS
- Test of the user experience: AttrakDiff

1.2.4. Outro (5 minutes)

2. Health professionals' interview guide

2.1. Introduction (10 minutes)

- About 1 hour with different subjects:
  - questions about the clinical practice and how PD symptoms are evaluated in the practice (introduction)
  - questions about what is known about BWS monitoring and the perception (topics # 1 and # 2)
  - questions about expectations regarding the technology (topic # 3)
- No right or wrong answer, no judgement: this is not an assessment, interest in personal opinion, the participant can be completely frank
- Recording of the conversation to be able to correctly transcribe what is said during the interview and to be able to exchange more freely: this recording will only be used within the purpose of this study and the information collected will remain anonymous.
- The participant is free to want to stop the recording or not answer a question, the participant can ask questions at any time

2.2. Questions

General introduction (5 minutes)

1. Would you say that you are satisfied with the technical means you have for patients' follow up in their PD?
2. Can you give your opinion on methods for monitoring the disease?
3. What would you like to change to improve your patient follow-up?
4. How would you describe your practice of new technologies? What is the place of this type of tool in your clinical practice?

Topic #1: Understanding and expectations (5 minutes)

1. What do you think of the use of new technologies to monitor chronic conditions?
2. Have you ever heard of BWS monitoring?
    a. If yes: what is your definition?
    b. If not: can you guess?
3. Is this the first time you've been involved in a project with BWS?
    a. If yes, what is your reaction?
    b. If not, what was your first impression?
4. Have you changed your perception of BWS monitoring? What is it today?

Presentation of the three devices and respective reports (5 minutes)

Topic #2: Perception of the follow up by monitoring with sensors (10 minutes)

1. What is your opinion on monitoring PD using technologies such as BWS?
2. How useful do you think BWS monitoring currently is for you?
3. Can you give some examples of situations that you think could benefit from BWS monitoring?
4. Can you give examples of cases where BWS monitoring might not be a good tool for PD follow up?
5. How do you think getting the patient to wear a BWS to record their symptoms will be:
    a. an advantage for your practice and/or for the patient?
    b. a disadvantage for your practice and/or for the patient?
6. Do you think of features you would like to see with disease monitoring tools?
7. Do you have anything to add?

Topic #3: Expectations and perspectives (10 minutes)

1. What are your expectations for BWS monitoring in general?
2. What value do you see in BWS monitoring?
3. Do you see a use rather oriented towards current practice? Can you give concrete cases?
4. Or research? Do you have any examples to give?
5. What impact do you feel from BWS monitoring on managing motor symptoms?
6. Do you think that the use of BWS could concern you?
7. Do you think that following the progress of your patients with this type of device will improve your working conditions? If yes, how? If not why?
8. What do you think you would use it for if you had this device for your practice?
9. Do you have any other ideas to add? Any other comments?

2.3. Outro (5 minutes)
